# Mitochondrial Protease ClpP: Cancer Marker and Drug Target

**DOI:** 10.3390/ph18101443

**Published:** 2025-09-25

**Authors:** Domenico Armenise, Olga Maria Baldelli, Anselma Liturri, Gianfranco Cavallaro, Cosimo Gianluca Fortuna, Savina Ferorelli, Morena Miciaccia, Maria Grazia Perrone, Antonio Scilimati

**Affiliations:** 1 Research Laboratory for Woman and Child Health, Department of Pharmacy-Pharmaceutical Sciences, University of Bari “Aldo Moro”, Via E. Orabona 4, 70125 Bari, Italy; domenico.armenise1@uniba.it (D.A.); olga.baldelli@uniba.it (O.M.B.); anselma.liturri@uniba.it (A.L.); savina.ferorelli@uniba.it (S.F.); antonio.scilimati@uniba.it (A.S.); 2 Laboratory of Molecular Modelling and Heterocyclic Compounds ModHet, Department of Chemical Sciences, University of Catania, Viale Andrea Doria 6, 95125 Catania, Italy; gianfranco.cavallaro@phd.unict.it (G.C.); cg.fortuna@unict.it (C.G.F.)

**Keywords:** human ClpP protease, quality control system, mitochondrial proteostasis, ClpP overexpression, ClpP activators/inhibitors, cancer

## Abstract

**Background**: The human mitochondrial ClpP is a serine protease located in the mitochondrial matrix responsible for degrading short lived regulatory proteins as well as misfolded or damaged proteins, thereby maintaining cellular homeostasis. Proteastasis dysregulation is linked to tumor progression. **Methods**: We conducted a literature review (2020–2025) using PubMed and Scopus, focusing on studies addressing ClpP structure, function, activity modulation, and cancer relevance. Keywords included “ClpP”, “ClpP activators”, “ClpP inhibitors”, and “mitochondrial protease”. **Results**: ClpP is upregulated in many tumors compared to normal tissues. Cancer cells depend on ClpP for mitochondrial proteostasis, metabolic adaptation, and survival. ClpP proteolytic activity modulation—via activators or inhibitors—disrupts these processes showing efficacy even in clinical setting. **Conclusions**: ClpP is emerging as a key player in cancer pathophysiology and holds potential as a therapeutic target. Its selective overexpression in tumors, along with its involvement in mitochondrial homeostasis, makes it a compelling candidate for precision oncology.

## 1. Introduction

Mitochondrial proteostasis has emerged as a central vulnerability in cancer biology, particularly in malignant neoplasms with high oxidative demands or defective quality control systems. Among mitochondrial surveillance proteins, the human caseinolytic protease P (*h*ClpP) has gained increasing attention as a therapeutic target in cancer investigations. Located in the mitochondrial matrix, *h*ClpP forms together with ClpX the complex ClpXP and plays a crucial role in degrading regulatory proteins and misfolded or damaged proteins, thereby preserving mitochondrial bioenergetics and integrity [1]. Under physiological conditions, ClpP acts as a housekeeping protease, maintaining protein quality control as part of the mitochondrial unfolded protein response (UPRmt). This system coordinates molecular chaperones (HSP60, HSP10, mtHSP70) and proteases (LONP1, ClpP) to restore mitochondrial function during stress [2]. By eliminating irreversibly damaged proteins, ClpP contributes to the stability of key pathways including oxidative phosphorylation (OXPHOS), mitochondrial translation, and intermediary metabolism. In cancer, however, this quality control machinery is often co-opted to support the survival of malignant cells. Tumors may exhibit elevated *h*ClpP expression, exploiting its proteolytic function to attenuate proteotoxic stress and support metabolic adaptation. This pathological dependence has spurred the development of compounds that target *h*ClpP, either through hyperactivation or inhibition. Recent investigations have found that hyperactivation, rather than inhibition, may induce catastrophic mitochondrial stress, rendering ClpP activity an attractive vulnerability in oncology [3,4,5,6,7]. Based on literature published between 2020 and 2025, this review provides a tumor-specific overview of *h*ClpP as a therapeutic target and its pharmacological activity modulation. Unlike previous reviews, which mainly emphasized ClpP structural biology, this review adopts also a distinct medicinal chemistry perspective: chemical structures of ClpP activators and inhibitors are described, while mechanistic insights and preclinical data are systematically organized by tumor type. The rationale for illustrating the structures of different modulators is to show how worldwide studies have attempted to affect ClpP activity using structurally diverse compounds. This comparative view not only highlights the translational potential of ClpP activity modulators but also aims to stimulate researchers working in different oncological areas to explore the role of ClpP within their tumor models and to inspire the design of novel compounds.

## 2. Results

### 2.1. ClpP and ClpX: Expression, Localization, and Prognostic Relevance in Human Cancers

**Figure 1:**
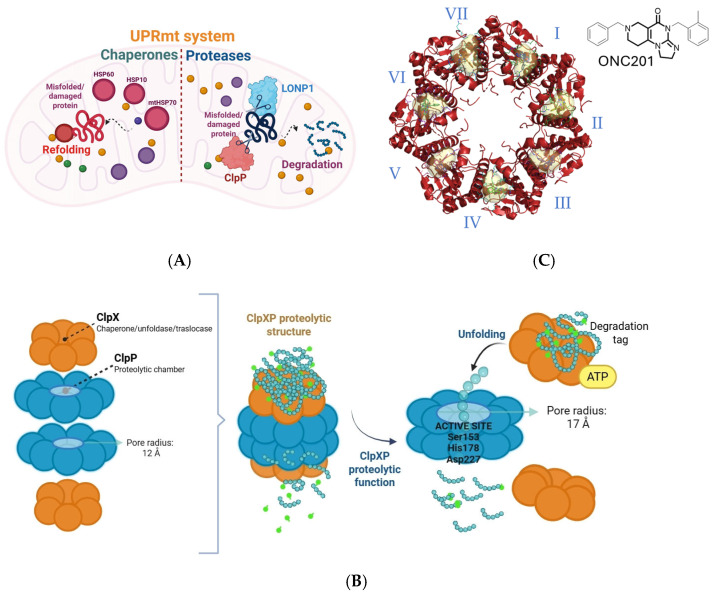
(**A**) Schematic representation of UPRmt system. Chaperones (HSP60, HSP10, mtHSP70) assist in refolding misfolded proteins, while proteases (LONP1, ClpP) degrade irreparably damaged proteins, thereby preserving mitochondrial proteostasis. (**B**) Schematic representation of the human ClpXP proteolytic complex. ClpX recognizes protein substrates via specific degradation tags and uses ATP hydrolysis to unfold them. The unfolded substrates are translocated into the proteolytic chamber of *h*ClpP, where the catalytic triad (Ser153, His178, Asp227) cleaves them into small peptides that are released through lateral pores. (**C**) Seven ONC201 molecules bind to the hydrophobic pockets between ClpP subunits, promoting stabilization of the active tetradecameric complex.

Human ClpP actively participates in protein homeostasis by forming the ClpXP complex with ClpX. Due to their central role in proteostasis, ClpP and ClpX also serve as effectors of the UPRmt, an evolutionarily conserved response that helps maintain mitochondrial and energetic homeostasis under stress conditions. Upon mitochondrial dysfunction, UPRmt induces the expression of chaperones and proteases that refold or degrade misfolded proteins, restoring mitochondrial function. UPRmt comprises both chaperone and protease components: chaperones such as HSP60, HSP10, and mtHSP70 facilitate protein refolding, while proteases including LONP1 and ClpP degrade irreparably damaged proteins that can not be rescued by chaperone activity (Figure 1A). Together, this system maintains mitochondrial integrity under continuous oxidative and proteotoxic stress [2]. To assemble the ClpXP complex, two *h*ClpP heptamers form a barrel-shaped tetradecamer capped at both ends by ClpX hexamers. This architecture creates a proteolytic chamber where unfolded substrates are degraded by the ClpP catalytic triad (Ser153, His178, Asp227). ClpX functions as a gatekeeper, recognizing substrates via degradation tags and using ATP hydrolysis to unfold and translocate them into ClpP. Substrates are then cleaved into small peptides (5–11 amino acids) and released through lateral pores [8] (Figure 1B). The substrate pool of ClpXP includes proteins of the Krebs cycle, OXPHOS, mitochondrial translation, and fatty acid/amino acid metabolism. ClpP can adopt three distinct conformational states: extended, compact, and compressed. These states are primarily distinguished by the architecture of the handle domains, which form a strand–turn–helix motif that interlocks the heptameric rings of the barrel. The relative orientation of the catalytic triad is directly coupled to the positioning of the handle domains. Only the extended tetradecameric state aligns the catalytic triad in a productive geometry and is catalytically active. By contrast, the compact and compressed states, characterized by shortened or kinked handle helices and disordered β-strands, misalign the catalytic triad and are inactive. Collectively, all known activators of ClpP act as allosteric modulators that decouple the structural regulation of the protease: they stabilize compact or compressed inactive states, promote axial pore opening, destabilize regulatory domains, and disrupt catalytic triade alignment. This combination drives mitochondrial proteostasis imbalance, leading to cellular stress and selective tumor cell death, becoming a promising strategy to collapse mitochondrial function. Structural studies show that seven ONC201, the most known ClpP activator, molecules bind within the hydrophobic pockets between adjacent ClpP subunits, enlarging the axial entrance pore from 12 to 17 Å, altering *N*-terminal dynamics, and modulating the catalytic triad position. This allows imipridones such as ONC201 and ONC212 to activate ClpP even in the absence of ClpX [9,10,11] (Figure 1C).

**Figure 2:**
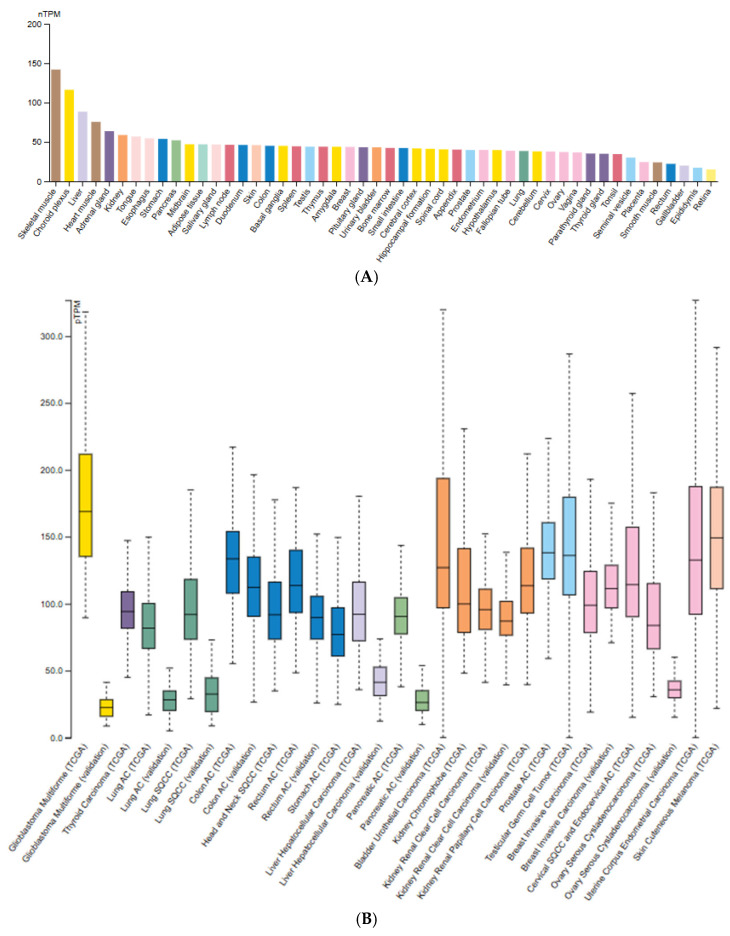
ClpP mRNA expression levels across normal (**A**) and tumor tissues (**B**); (**A**) Expression of ClpP in normal human tissues based on the Consensus dataset (v22, Human Protein Atlas), integrating RNA-seq data from GTEx, HPA, and FANTOM5. Values are reported as normalized transcripts per million (nTPM); (**B**) ClpP expression across various cancer types from TCGA RNA-seq dataset. Figures adapted from the Protein Atlas Database (v22.proteinatlas.org).

As interest in ClpP as a drug target grows, expression patterns provide crucial context. According to the Human Protein Atlas, ClpP is ubiquitously expressed across normal human tissues, with highest levels in metabolically active organs such as skeletal muscle, heart, and liver (Figure 2A) [12]. This profile is consistent with its role as a housekeeping protease. The Cancer Genome Atlas (TCGA) tumor data, however, reveal heterogeneous expression, with the highest levels in glioblastoma multiforme (GBM), bladder carcinoma, testicular germ cell tumors, endocervical carcinoma, and melanoma, whereas lung, pancreatic, and ovarian cancers display consistently low expression (Figure 2B).

Across tumor types such as colon, breast, and prostate, ClpP expression extent is medium to low and non–tumor specific but consistently detected [13,14]. Immunohistochemistry confirms granular cytoplasmic staining consistent with mitochondrial localization of ClpXP [15]. Prognostic analyses show no significant correlation between ClpP expression and overall survival in cancers such as melanoma or glioma, indicating a primarily functional rather than predictive role [12].

**Figure 3:**
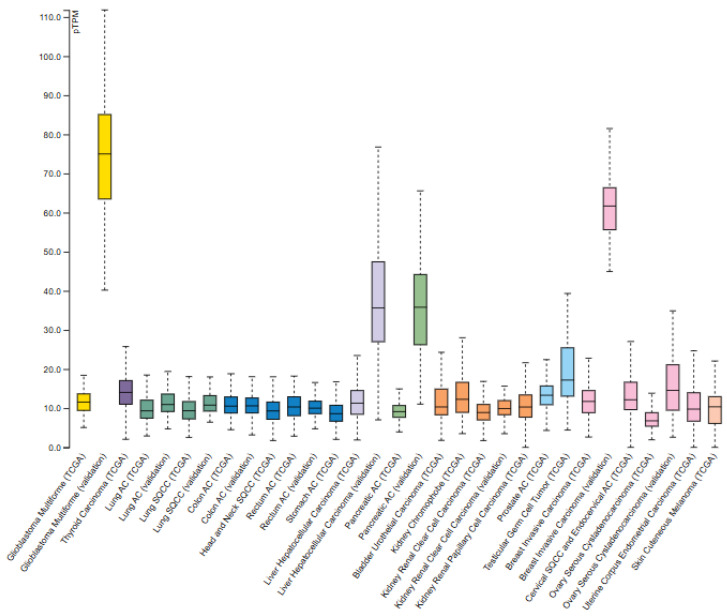
ClpX mRNA expression in normal and tumor tissues [Protein Atlas Database]. ClpX expression in tumors based on RNA-seq data from The Cancer Genome Atlas (TCGA). Figure adapted from the Protein Atlas Database (v22.proteinatlas.org).

Similarly, ClpX is ubiquitously expressed at moderate-to-high levels across tumors (Figure 3). Pathology Atlas data indicates both cytoplasmic and nucleoplasmic localization overlapping with ClpP [15]. Like ClpP, ClpX expression is not prognostic in any analyzed cancer type, reinforcing its role as a conserved component of mitochondrial proteostasis [7].

In summary, ClpP and ClpX are stable and ubiquitously expressed to varying degrees in human healthy tissues and cancers, supporting their value as therapeutic targets in malignancies with heightened mitochondrial stress or metabolic vulnerability [13].

### 2.2. ClpP-Targeting Compounds: Therapeutic Relevance and Stage of Development

Over the past few years, a growing number of structurally diverse *h*ClpP activators have been identified, distinguished primarily by variations in their central core ring systems such as ONC201, IMP075, NCA029, ONC206, ONC212, TR57, ZG36, ZK53, 7k, ClpP-1071 and THX6 (Table 1). These compounds hyperactivate *h*ClpP, triggering uncontrolled proteolysis of respiratory chain subunits, mitochondrial ribosomal (mitoribosome) proteins, and key metabolic enzymes [2,3,4,5,6,7]. This proteostatic collapse leads to disruption of OXPHOS, accumulation of reactive oxygen species (ROS), and induction of apoptosis via the ATF4/CHOP stress response pathway [8,16,17].

In contrast, research on ClpP inhibitors has been relatively limited. Here, we highlight two representative compounds, A2-32-01 and TG53, subsequently used in advanced vitro studies (Table 1).

ClpP activators bind to hydrophobic pockets located at the interfaces between the subunits of the tetradecamer (Figure 1B), in a region spatially distinct and distant from the catalytic site. This allosteric binding induces conformational rearrangements of the protease. A hallmark feature is the widening of the axial pore, which shifts from the closed configuration typical of the apo-state to an open and more permissive channel, thereby enhancing substrate accessibility. Concomitantly, the handle domains lose part of their ordered secondary structure and become shortened, while the normally flexible *N*-terminal loops move outward from the central axis, further contributing to pore opening [11].

A second key effect of activator binding is the perturbation of the catalytic triad (Ser153, His178, Asp227), which becomes misaligned and catalytically inactive. Thus, in the presence of activators, ClpP adopts a conformation that facilitates substrate entry, but compromises its catalytic activity, resulting in uncontrolled protein degradation [11]. At the molecular level, ligand-protein interactions involve hydrophobic contacts, aromatic stacking, hydrogen bonds, and anion–π interactions. These stabilizing forces maintain ClpP in its “open” yet inactive conformation.

The therapeutic potential of *h*ClpP activation has been demonstrated across multiple tumor types. In acute myeloid leukemia (AML), ClpP activators can destroy leukemic stem cells and help defeat resistance to BCL-2 inhibitors [9,16,20,21,22]. In colorectal cancer, compounds such as ONC201 and IMP075 impair mitochondrial metabolism and induce apoptosis [3]. In prostate cancer, ONC201 is active in neuroendocrine differentiated tumors, although resistance may arise via lineage plasticity [23]. Gastric carcinoma shows differential ClpP expression associated with mitochondrial stress and carcinogenesis [24]. Pancreatic cancer cells respond to ONC201 through activation of the integrated stress response (ISR) induction and mitochondrial collapse [25]. In hepatocellular carcinoma (HCC), restoration of *h*ClpP function improves steatohepatitis and reduces tumor development and progression [26]. In non-small cell lung cancer (NSCLC), ClpP activators like ZK53 collapse mitochondrial respiration and induce p53-independent cell death [7]. In breast cancer, ClpP activation sensitizes cells to Tumor Necrosis Factor-Related Apoptosis-Inducing Ligand (TRAIL) and enhances apoptotic signaling, particularly in HER2+ subtypes [27]. In ovarian cancer, ClpP is stabilized by HSPA8, a chaperone protein belonging to the HSP70 family, and contributes to cisplatin resistance, indicating a potential role in the regulation of chemoresistance [28]. In brain tumors, especially H3K27-altered diffuse midline glioma (DMG H3K27-altered), ONC201 induces ClpP dependent metabolic stress and is under evaluation in clinical trials such as the recruiting PNOC022 [29].

### 2.3. ClpP Modulation in Acute Myeloid Leukemia (AML)

#### 2.3.1. Mechanism of Action

Mitochondrial metabolism has emerged as a crucial determinant of leukemic cell survival, drug resistance, and stemness, particularly in AML and chronic lymphocytic leukemia (CLL). Recent studies have validated ClpP as both a genetic vulnerability and a pharmacological target in leukemia, with evidence supporting its dual therapeutic modulation, either by hyperactivation or inhibition, as both approaches ultimately collapse mitochondrial integrity and induce leukemic cell death. Ishizawa et al. reported that the 45% of 511 primary AML samples analyzed showed the overexpression of ClpP, associated with mitochondrial unfolded protein response activation [9]. Hyperactivation of ClpP by ONC201 or its analogs ONC206/ONC212 induces unregulated proteolysis of mitochondrial respiratory complexes, leading to ATP depletion, increased ROS, and apoptosis of AML cells, including TP53-mutant subtypes, without harming normal hematopoietic progenitors [9]. Similarly, Mirali and Schimmer identified ClpP as one of the top mitochondrial dependencies in AML using large-scale short hairpin RNA (shRNA) screening. ClpP knockdown impaired complex II activity and leukemic stem cell engraftment while sparing normal CD34^+^ cells [30].

#### 2.3.2. Preclinical Data

ClpP activators ZG36 and 7k (Table 1) have shown enhanced both potency and pharmacokinetics. ZG36, a diacylpyrimidine-based compound, induces mitochondrial collapse and loss of mtDNA in AML models [6] while 7k, obtained via ring-opening strategies, displays superior ClpP engagement and apoptotic induction in vitro and in vivo [16]. Conversely, ClpP inhibition has emerged as an alternative strategy to disrupt mitochondrial proteostasis. A2-32-01 and TG53 (Table 1) covalently bind the catalytic serine of ClpP, impairing respiratory chain function and inducing leukemic cell death [18,19,20]. Despite differing mechanisms, both activation and inhibition of ClpP converge on mitochondrial collapse as a shared cytotoxic outcome. Notably, ClpP activation has shown synergy with venetoclax (VEN)-based therapies [31]. A recent study revealed that ONC201 resensitizes AML cells to VEN + cytarabine (AraC) by destabilizing oxidative phosphorylation and mitochondrial Ca^2+^ homeostasis, mechanisms implicated in minimal residual disease [32]. These effects were particularly pronounced in BCL2-dependent AML, highlighting the value of ClpP activators in overcoming resistance. Feng et al. demonstrated that serine phosphorylation acts as a mitochondrial degron, promoting ClpXP-mediated degradation of respiratory chain subunits. ONC201-induced hyperactivation bypasses substrate specificity, resulting in broad, uncontrolled proteolysis and cell death [33].

#### 2.3.3. Clinical Outlook

**Figure 4:**
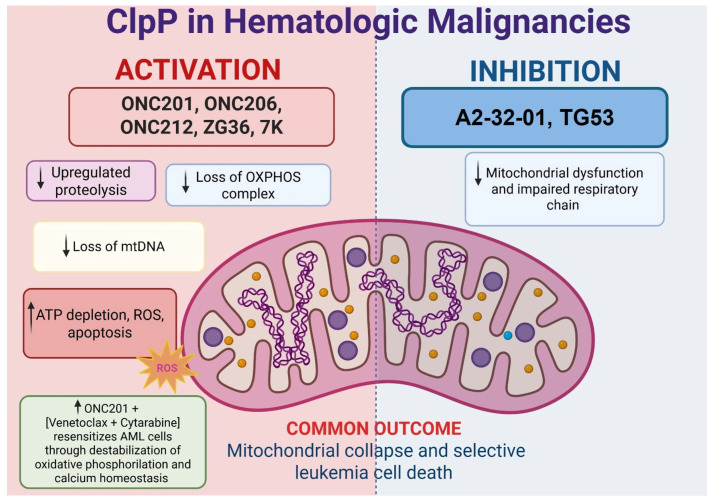
Both pharmacological activation and inhibition of ClpP converge on mitochondrial collapse and leukemic cell death. ClpP activators such as ONC201, ONC206, ONC212, ZG36, and 7k induce unregulated proteolysis, loss of OXPHOS complexes, mtDNA depletion, ATP loss, ROS accumulation, and apoptosis. Inhibition with A2-32-01 and TG53 disrupts respiratory chain function, also leading to mitochondrial dysfunction. Notably, ClpP activation synergizes with venetoclax plus cytarabine by destabilizing oxidative phosphorylation and calcium homeostasis. Despite distinct mechanisms, both strategies selectively impair leukemia survival.

The therapeutic relevance of ClpP also extends to CLL. Fatima et al. demonstrated that ONC212 impairs BCR and BCL-2 signaling, induces ATF4 expression, and disrupts microenvironmental survival cues in CLL cells, including TP53-deficient models, supporting its potential application in lymphoid malignancies [34]. Collectively, this evidence establishes ClpP as a master regulator of mitochondrial proteostasis and a promising therapeutic target in both myeloid and lymphoid malignancies (Figure 4).

### 2.4. Solid Tumors with High Mitochondrial Metabolism

#### 2.4.1. ClpP Modulation in Pancreatic Ductal Adenocarcinoma (PDAC)

##### Mechanism of Action

Pancreatic ductal adenocarcinoma (PDAC) is one of the most lethal solid tumors, with a five-year survival rate below 10% despite ongoing investigations. Its clinical aggressiveness is driven by profound chemoresistance and near-universal KRAS mutations, which reprogram tumor metabolism toward mitochondrial respiration, redox adaptation, and anabolic biosynthesis. This metabolic dependency renders mitochondria a critical vulnerability in PDAC. Among mitochondrial targets, ClpP has recently gained attention.

Wang et al. characterized ZG111 (lead compound of ZG36), a ClpP activator capable of inducing mitochondrial dysfunction and tumor suppression in vitro and in vivo [25]. ZG111 binds ClpP and aberrantly activates its proteolytic activity, triggering unregulated degradation of electron transport chain (ETC) components. This results in mitochondrial membrane potential collapse, OXPHOS inhibition, and ROS accumulation. Subsequent activation of the JNK/c-Jun and endoplasmic reticulum (ER) stress pathways culminate in cell cycle arrest and apoptosis. Notably, ZG111 exhibited efficacy in PDAC models regardless of KRAS mutational status, indicating that ClpP activation can bypass classical oncogenic dependencies. Moreover, ClpP expression positively correlated with patient survival, supporting its dual role as both a therapeutic target and a prognostic biomarker.

##### Preclinical Data

Supportive evidence was provided by Ferrarini et al., who demonstrated that ONC212 promotes ClpP-mediated mitochondrial dysfunction in PDAC cells [35]. This effect involves the disruption of mitochondrial ATP production, degradation of ClpX, and induction of apoptosis in OXPHOS-dependent cell lines. Glycolytic cells exhibited growth arrest but became more sensitive to apoptosis upon co-treatment with 2-deoxy-D-glucose (2-DG), highlighting the importance of metabolic context in determining the efficacy of ClpP activators. Additionally, ONC212 engaged AMP-activated protein kinase (AMPK) and mitochondrial stress signaling, revealing dual outcomes: stress adaptation in some settings and energy collapse in others.

Czuczi et al. extended this strategy by designing ferrocene-imipridone with enhanced ROS generation [36]. Although slightly less potent than ONC201, the compounds reported exhibit strong long-term cytotoxicity and selectivity for tumor cells over normal cells, supporting a dual mechanism of action involving ClpP activation and ROS accumulation.

##### Clinical Outlook

**Figure 5:**
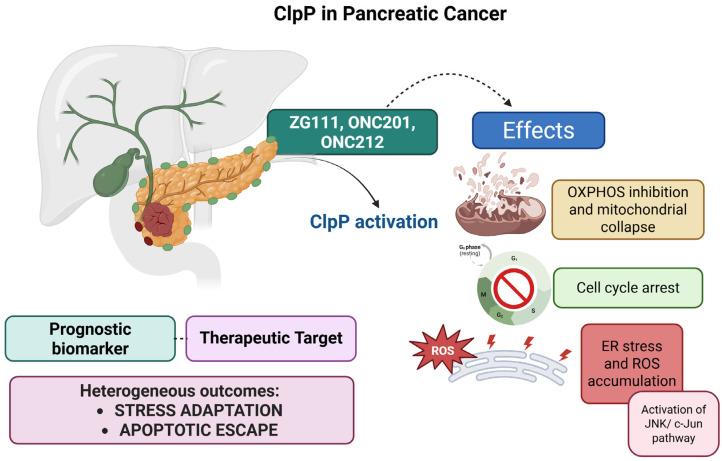
Pharmacological activation of ClpP by ZG111, ONC201, and ONC212 induces mitochondrial dysfunction in PDAC. Mechanistic effects include inhibition of OXPHOS, mitochondrial collapse, ATP depletion, cell cycle arrest, and ROS accumulation associated with ER stress and activation of the JNK/c-Jun pathway. ClpP expression also correlates with patient overall survival, highlighting its dual role as a prognostic biomarker and therapeutic target.

A potential limitation to ClpP-based therapies was highlighted by Zhang et al., who showed that dopamine signaling can impair imipridone efficacy in PDAC [37]. Dopamine pre-treatment, but not acute co-treatment, reduced the cytotoxic effects of ONC201, ONC206, and ONC212, particularly in PDAC and colorectal cancer cells. This effect was not mediated by classical DRD2 agonism (e.g., sumanirole) and did not interfere with ClpP activation or ISR induction. Instead, dopamine upregulated anti-apoptotic proteins such as p-AKT, p-Bad, XIAP, and cFLIP, thereby promoting survival signaling downstream mitochondrial stress. These findings suggest that extrinsic neurotransmitter signals may modulate the efficacy of ClpP targeting therapies in a context-dependent manner. Collectively, these studies define ClpP as a central vulnerability in PDAC. However, the heterogeneous cellular responses to ClpP activation, ranging from stress adaptation to apoptotic escape, highlight the need for refined biomarker-based patient stratification (Figure 5).

Future development should prioritize the validation of ClpP expression and metabolic dependency biomarkers in PDAC samples, along with efforts to map ClpP substrates and elucidate their downstream signaling effects. In addition, optimizing therapeutic combination strategies will be essential, while simultaneously investigating potential resistance mechanisms mediated by catecholamine signaling or microenvironmental influences.

### 2.5. ClpP in Liver Cancer: From Mitochondrial Stress Regulator to Therapeutic Target

#### 2.5.1. Mechanism of Action

First characterized in microbial systems and neurodegenerative diseases, ClpP is implicated in hepatic disorders such as non-alcoholic steatohepatitis (NASH) to hepatocellular carcinoma (HCC), and liver metastases. Choi et al. demonstrated that ClpP deficiency promotes the progression of NASH, a major risk factor for HCC [38]. In high-fat/high-fructose diet of fed mice, ClpP expression was significantly reduced, leading to impaired fatty acid oxidation (FAO), ATP depletion, and ROS accumulation. These mitochondrial defects activated the cGAS–STING–TBK1 inflammatory axis and contributed to steatosis, fibrosis, and hepatocellular injury. Restoring ClpP levels, either through gene overexpression or treatment with the ClpP activator A54556A, normalized FAO, reduced oxidative stress and inflammation, and improved liver histology. These results indicate that ClpP downregulation in steatotic liver is not only a biomarker of mitochondrial dysfunction but also a contributing factor, promoting oncogenic transformation through redox imbalance and metabolic damage.

#### 2.5.2. Preclinical Data

Cao et al. extended this model by demonstrating that ClpP hyperactivation selectively induces apoptosis in established HCC cell lines [26]. Treatment with ONC206 triggers mitochondrial swelling, membrane depolarization, ATP depletion, and ROS accumulation, ultimately resulting in mitochondrial collapse and cell death. Notably, ClpP knockdown abolished ONC206-induced cytotoxicity, confirming ClpP as its primary molecular target. Proteomic analysis revealed degradation of ETC subunits, including succinate dehydrogenase A (SDHA) and succinate dehydrogenase B (SDHB). Moreover, pharmacological or genetic inhibition of autophagy (e.g., ATG5 silencing or chloroquine treatment), further enhanced ONC206 efficacy, highlighting the compensatory role of autophagy in the mitochondrial stress response and supporting combined therapeutic approaches.

Extending these insights to metastatic liver disease, Chattopadhyay et al. evaluated ClpP-targeting imipridones in orthotopic models of uveal melanoma liver metastases [39], which are highly reliant on mitochondrial metabolism. ONC201 and ONC212 reduced tumor burden and prolonged survival in vivo. ONC212 disrupted amino acid metabolism, depleted glutathione and polyamines, and increased mitochondrial ROS. These changes impaired the Warburg effect, suppressed protein synthesis, and triggered lipid peroxidation and apoptotic signaling. High ClpP expression in uveal melanoma tumors was correlated with increased treatment sensitivity, highlighting its potential as a predictive biomarker. The unique metabolic environment of the liver may amplify ClpP-mediated stress, making liver-dominant tumors particularly vulnerable to ClpP-targeted strategies.

#### 2.5.3. Clinical Outlook

**Figure 6:**
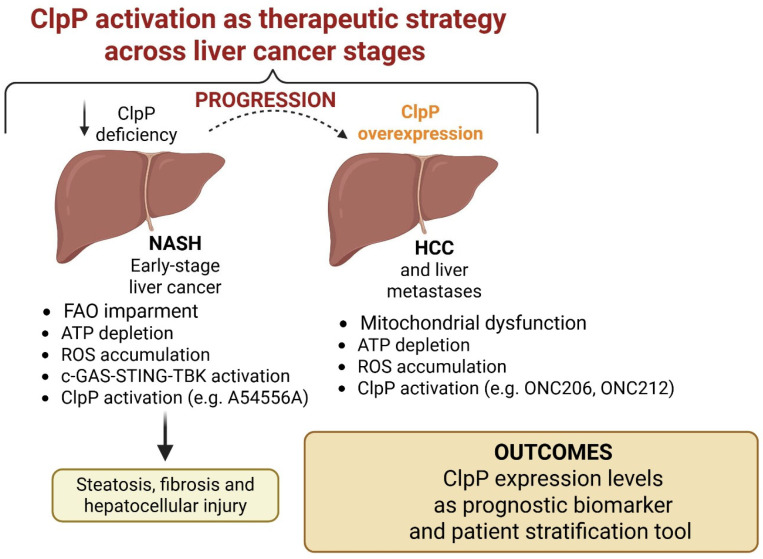
In early liver disease such as NASH, ClpP deficiency leads to impaired fatty acid oxidation, ATP depletion, ROS accumulation, and activation of the cGAS–STING–TBK1 inflammatory axis, promoting steatosis, fibrosis, and hepatocellular injury. During progression to HCC and liver metastases, ClpP becomes overexpressed, supporting mitochondrial dysfunction, ATP depletion, and ROS accumulation. Pharmacological activation of ClpP by ONC206 or ONC212 induces mitochondrial collapse and tumor cell death.

Collectively, these studies support a stage-dependent model for ClpP function in hepatocarcinoma. In early stages, such as NASH, loss of ClpP contributes to mitochondrial dysfunction and chronic inflammation, creating a pro-tumorigenic environment [33]. In contrast, in advanced HCC and metastatic disease, ClpP is frequently overexpressed and represents a therapeutic vulnerability that can be targeted by small-molecule activators such as ONC206 and ONC212 (Figure 6) [26,39].

This evolving model carries key implications. First, ClpP expression levels may serve as both a prognostic biomarker and patient stratification tool for ClpP-directed therapy. Second, combination regimens involving ClpP activators and autophagy or redox inhibitors may enhance efficacy. Third, early ClpP restoration in high-risk metabolic liver disease could offer a chemo-preventive approach. Finally, a deeper understanding of the regulatory networks, substrates, and stress-adaptive role of ClpP will be critical to translate preclinical results into clinical benefits in primary and secondary liver malignancies. In conclusion, ClpP acts as both a guardian and a potential driver in liver disease progression. Targeting ClpP may offer a novel therapeutic strategy for both preventing tumor development and eliminating established malignancies, addressing the full spectrum of liver pathology.

### 2.6. ClpP in Gastric Cancer: Molecular Alterations and Therapeutic Targeting

#### 2.6.1. Mechanism of Action

Proteomic profiling of human gastric carcinoma has revealed altered expression of mitochondrial ClpP, indicating its potential contribution to gastric tumorigenesis. In a landmark study, ClpP was identified among a subset of deregulated mitochondrial enzymes, alongside cytochrome c oxidase subunit 5A (COX5A) and enoyl-CoA hydratase 1 (ECH1), suggesting impairments in redox homeostasis, mitochondrial protein quality control, and metabolic rewiring in tumor cells [40]. Additionally, dysregulated ClpP expression was observed in conjunction with mitotic checkpoint regulators mitotic arrest deficient 1 (MAD1L1) and end binding 1 (EB1) and stress-response proteins (HSP27, CYR61), highlighting a broader network linking mitochondrial dysfunction to chromosomal instability and resistance to apoptosis in gastric cancer.

#### 2.6.2. Preclinical Data

In the gastric adenocarcinoma cell lines AGS, SNU-1, SNU-5, and SNU-16, ONC201 significantly increased DR5 expression and sensitized cells to recombinant human TRAIL (rhTRAIL) or its PEGylated trimeric form, TLY012, resulting in strong activation of the extrinsic apoptotic pathway via caspase-8 and -3 [24].

**Figure 7:**
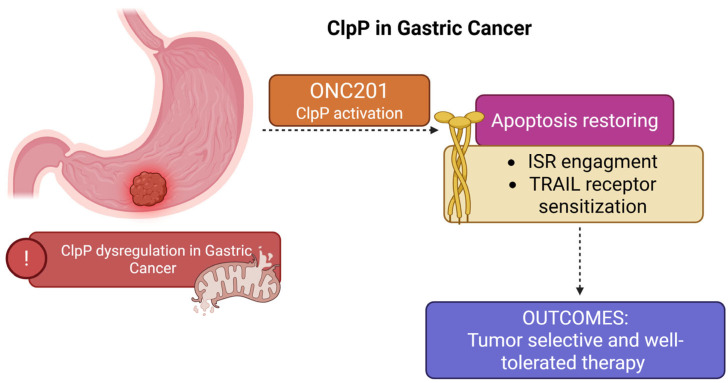
Dysregulation of ClpP contributes to mitochondrial dysfunction and impaired apoptotic signaling in gastric carcinoma. ClpP activation by ONC201 restores apoptosis through engagement of the ISR and sensitization of TRAIL receptors, resulting in a tumor-selective and well-tolerated therapeutic effect.

Notably, this synergistic interaction between ONC201 and TRAIL was associated with increased sub-G1 accumulation, Poly (ADP-ribose) polymerase (PARP) cleavage, and downregulation of anti-apoptotic proteins including cIAP1, XIAP, and cFLIP. The apoptotic response was consistent across most gastric cancer models, except for partially resistant AGS and SNU-16 cell lines, suggesting that tumor heterogeneity affects ONC201 responsiveness., the in vivo treatment with ONC201 and TLY012 inhibited tumor growth in AGS and SNU-1 xenografts and induced apoptosis in gastric cancer organoids, without signs of systemic toxicity [24,41]. Although performed in immunodeficient mice and limited to a defined panel of models, these results confirm functional involvement of ClpP in ISR-driven apoptosis in gastric cancer (Figure 7).

#### 2.6.3. Clinical Outlook

ClpP dysregulation in gastric adenocarcinoma underscores a broader landscape of mitochondrial dysfunction and highlights *h*ClpP as a compelling therapeutic target. Pharmacological activation of ClpP by ONC201 restores apoptotic signaling through ISR engagement and TRAIL receptor sensitization, providing a tumor-selective and well-tolerated strategy for gastric cancer treatment. These results warrant further clinical investigation, particularly in gastric cancer subtypes characterized by mitochondrial dependence or intrinsic resistance to apoptosis, where ClpP-targeted therapies may provide significant benefits.

### 2.7. ClpP in Colorectal Cancer

ClpP expression has been found also in colorectal cancer (CRC), a malignancy characterized by high metabolic plasticity and frequent resistance to conventional chemotherapy.

Zhang et al. developed IMP075, an analog of ONC201 with improved mitochondrial affinity and bioavailability [3]. In HCT116 CRC cells, IMP075 shown potent cytotoxicity through ClpP activation, leading mitochondrial membrane depolarization, ATP depletion, increase in ROS levels, ultimately inducing ISR and apoptosis. Structural and biochemical analyses confirmed its tighter binding to ClpP (K_d_ = 0.25 μM vs. 26.8 μM of ONC201) and superior in vivo performance in xenograft models, alongside favorable pharmacokinetics and a reduced risk of cardiotoxicity [3].

**Figure 8:**
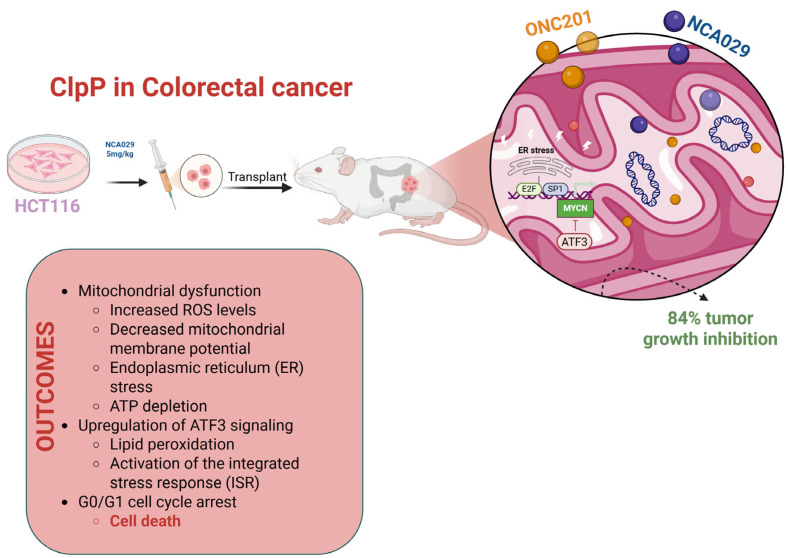
Schematic representation of the effects of ONC201 and NCA029 in HCT116 colorectal cancer models. Treatment induces mitochondrial dysfunction characterized by increased ROS levels, decreased mitochondrial membrane potential, ATP depletion, and ER stress. These alterations lead to upregulation of ATF3 signaling, lipid peroxidation, activation of the ISR, and G0/G1 cell cycle arrest, culminating in cell death. In xenograft models, NCA029 treatment resulted in 84% tumor growth inhibition, highlighting the therapeutic potential of ClpP-targeting compounds in colorectal cancer.

Further progress was achieved by Zhang and his coworkers with NCA029, a rationally designed ClpP activator derived from CCG1423, exhibiting high selectivity and potent activity against HCT116 cells with an IC_50_ of 1.0 μM. Molecular docking and mutagenesis confirmed stable interactions with TYR118, TYR138, and TRP146 in the hydrophobic pocket of ClpP. Functionally, NCA029 induced mitochondrial dysfunction, ROS generation, ISR activation, and transcriptional changes in stress and apoptotic pathways. The cytotoxic effects of NCA029 were strictly ClpP-dependent, exhibiting enhanced potency in ClpP-overexpressing cells and complete loss of activity in ClpP knockdown models. Specifically, NCA029 lacked antibacterial activity, thereby preserving the gut microbiota, and demonstrated excellent efficacy and tolerability in vivo [4]. These findings identify ClpP as a crucial metabolic checkpoint in CRC. Future clinical translation will benefit from biomarker-guided patient stratification and rational drug combinations exploiting the central role of ClpP in mitochondrial homeostasis and tumor survival (Figure 8).

### 2.8. ClpP in Prostate Cancer

#### 2.8.1. Mechanism of Action

Prostate cancer (PCa) represents a major cause of cancer-related death in men, with progression to castration-resistant prostate cancer (CRPC) and neuroendocrine prostate cancer (NEPC) posing significant therapeutic challenges. NEPC is particularly aggressive and characterized by loss of androgen receptor (AR) signaling, lineage plasticity, and treatment resistance.

Purcell et al. explored the impact of neuroendocrine differentiation (NED), responsible for increased tumor aggressiveness, resistance to treatments, and poor prognosis, on the sensitivity of prostate cancer cells to ClpP-activating imipridones. Using inducible expression of SOX2 and POU3F2 in LNCaP and DU145 cell lines, it was observed that although ClpP expression increased under prolonged SOX2 induction, this did not enhance sensitivity to ONC201 or its analogs such as ONC206 [42]. Indeed, only a modest resistance phenotype was observed, indicating that ClpP upregulation alone is not sufficient to predict sensitivity to ClpP modulators. These findings suggest that therapeutic efficacy is affected not only by ClpP levels, but also by the broader mitochondrial context and the cellular lineage or differentiation state, such as neuroendocrine features, which may modulate dependence on mitochondrial proteostasis [42].

#### 2.8.2. Preclinical Data and Clinical Outlook

Lee et al. demonstrated that ClpP and LONP1 cooperatively regulate mitochondrial proteostasis in PCa. Dual suppression of these proteases led to mitochondrial protein aggregation, impaired OXPHOS, increased ROS, and reduction in proliferative capacity. The co-expression of ClpP and LONP1 in prostate tumors, both located on chromosome 19q13, suggests a coordinated oncogenic stress–adaptive axis [43].

**Figure 9:**
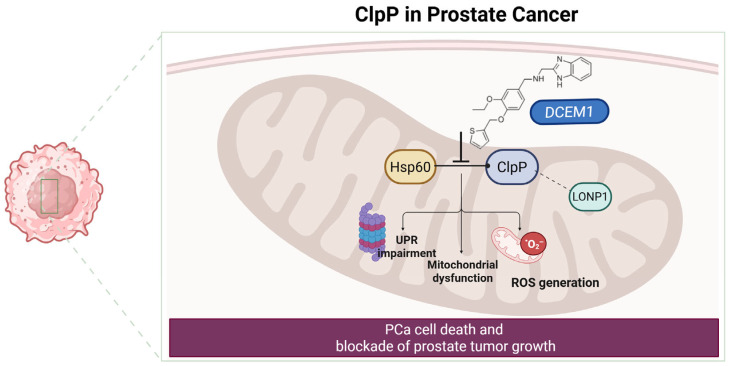
Schematic representation of the role of ClpP in PCa. HSP60 regulates ClpP expression via c-Myc, while LONP1 cooperates with ClpP to maintain mitochondrial proteostasis. Inibition of UPRmt by DCEM1 disrupts the HSP60-ClpP axis, leading to mitochondrial dysfunction, impaired proteostasis, and increased ROS generation. These effects culminate in PCa cell death and inhibition of tumor growth.

Kumar et al. demonstrated that the mitochondrial UPRmt is a key survival mechanism in PCa [44]. HSP60 transcriptionally regulates ClpP expression via c-Myc, meaning that HSP60 can activate ClpP production by stimulating the transcription factor c-Myc, which enhances ClpP gene transcription. When the interaction between HSP60 and ClpP is disrupted by an inhibitor of the UPRmt, such as DCEM1 (Figure 9), the mitochondrial protein quality control system becomes dysregulated. This leads to impaired mitochondrial bioenergetics, disruption of cellular adaptation signals (mitohormesis), and ultimately to cancer cell death and inhibition of PCa growth [44].

Thus, ClpP operates within a broader proteostasis network involving HSP60 and LONP1 and is regulated by oncogenic signaling pathways such as c-Myc, particularly in treatment-resistant PCa subtypes (Figure 9).

### 2.9. ClpP in Lung Cancer

#### 2.9.1. Mechanism of Action

A growing body of evidence recognized the crucial role of ClpP in lung cancer. It functions not only as a marker of mitochondrial stress but also as a driver of metabolic reprogramming, ferroptosis sensitivity, and therapeutic response in both non-small cell lung cancer (NSCLC) and small cell lung carcinoma (SCLC).

Genetic studies suggested that polymorphisms in the ClpP gene may contribute to lung cancer susceptibility. Li et al. identified two intronic single nucleotide polymorphisms (SNPs), rs10420388 and rs10418574, that were significantly associated with advanced-stage NSCLC and an increased risk of squamous cell carcinoma (SCC) [45]. These SNPs, predicted to be in regulatory regions, were absent in early-stage of the disease and may promote ClpP transcription, and elevate ClpP expression. Since high ClpP levels have previously been correlated with poor metastasis-free survival in NSCLC, these SNPs may contribute to a more aggressive disease phenotype by enabling cancer cells to better withstand metabolic and oxidative stress through enhanced mitochondrial proteolysis.

#### 2.9.2. Preclinical Data

Zhou et al. developed the activator of *h*ClpP ZK53 [7] that in lung squamous cell carcinoma (LUSC) models, promoted ClpP-mediated degradation of ETC proteins, thereby disrupting OXPHOS, inducing energetic stress, and blocking G1 cell cycle phase. ZK53 did not induce apoptosis but activated the ATM-mediated DNA damage response (DDR), linking mitochondrial dysfunction with nuclear genome surveillance. No correlation was observed between basal ClpP expression and sensitivity to ZK53, suggesting that mitochondrial dependency and downstream signaling context better predict therapeutic efficacy.

Ding et al. reported that ONC201 induced mitochondrial collapse and apoptosis across different SCLC models, regardless of their neuroendocrine differentiation status [46]. ONC201 cytotoxicity was particularly potent in the H1417 SCLC cell line, implicating mitochondrial proteotoxic stress as a universal vulnerability in this tumor subtype. While ONC201 exerts poly-pharmacological effects, its ClpP-dependent mechanism has been validated in other systems, suggesting similar involvement in lung neuroendocrine cancers.

Li et al. demonstrated that acute arsenic exposure in human pulmonary epithelial cells induced mtROS accumulation, membrane potential collapse, and ferroptotic death, alongside increased expression of ClpP and mtHSP70 [47]. These findings underscore ClpP role in responding to organellar stress and suggest a link between environmental toxins, mitochondrial damage, and potential carcinogenic transformation through ClpP-mediated adaptation.

#### 2.9.3. Clinical Outlook

**Figure 10:**
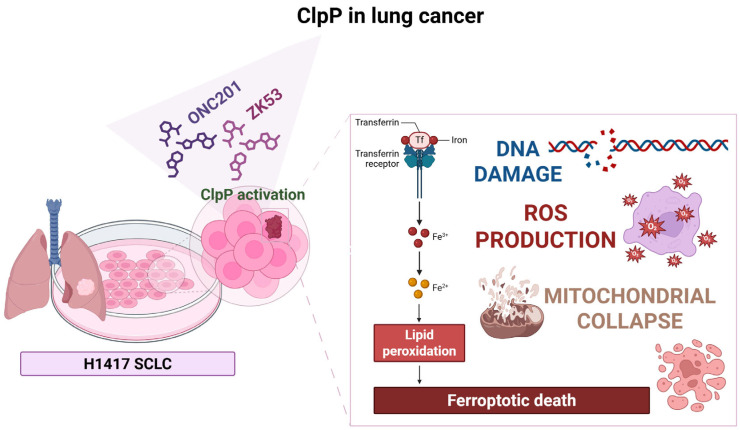
ClpP activation by ONC201 and ZK53 in H1417 SCLC induce mitochondrial dysfunction and excessive ROS production. These effects promote mitochondrial collapse, lipid peroxidation, and DNA damage, ultimately driving ferroptotic cell death.

In NSCLC, ClpP SNPs and ClpP overexpression may promote tumor progression by enhancing metabolic resilience. In LUSC and SCLC, ClpP activation by ZK53 or ONC201 disrupts mitochondrial proteostasis and energy production, resulting in cytostasis or apoptosis, depending on tumor subtype and metabolic context (Figure 10). Thus, ClpP represents a promising target for precision oncology in lung malignancies, integrating genetic, metabolic, and environmental signals into a druggable mitochondrial vulnerability.

### 2.10. Female Specific Tumors with Chemoresistance

#### ClpP in Ovarian Cancer: Targeting Mitochondrial Proteostasis to Overcome Chemoresistance

Epithelial ovarian cancer (EOC) remains one of the most lethal gynecologic malignancies, largely due to late-stage diagnosis and the frequent development of resistance to platinum-based chemotherapy [48,49,50]. Recent evidence has underscored the importance of mitochondrial dysfunction and proteostasis in chemotherapy response, highlighting the mitochondrial matrix protease ClpP as a promising therapeutic target.

Kou et al. found that ClpP expression is significantly reduced in cisplatin-resistant EOC cell lines compared to their parental counterparts [51]. Silencing ClpP in wild-type cells increased cisplatin IC_50_ values, whereas ClpP overexpression in resistant cells restored sensitivity to the drug. ClpP depletion promoted mitophagy through upregulation of PINK1, Parkin, and LC3-II, enhancing the clearance of damaged mitochondria and cell survival under treatment stress. Moreover, the chaperone HSPA8 was shown to destabilize ClpP, contributing to cisplatin resistance through enhanced mitophagy, suggesting a novel regulatory axis that may be leveraged to overcome chemoresistance in EOC [28].

Moreover, Kou et al. demonstrated that ClpP levels are lower in EOC cells than in normal ovarian epithelial cells and that its suppression promotes tumor cell proliferation, migration, and invasion [51]. Treatment with ONC201 induced mitochondrial dysfunction by degrading key electron transport chain components (NDUFA12, SDHA, SDHB), leading to mitochondrial membrane depolarization, ATP depletion, and caspase-3 activation. These pro-apoptotic effects occurred independently of p53 status and were accompanied by reduced motility and inhibition of epithelial–mesenchymal transition (EMT), linking ClpP activation to anti-metastatic activity.

Fan et al. demonstrated that ONC201 induces ClpP-mediated mitochondrial and endoplasmic reticulum (ER) stress in both EOC cell lines and KpB transgenic mouse models [52]. ONC201 also downregulated survival pathways (PI3K/AKT/mTOR, MAPK/ERK), reduced Vascular Endothelial Growth Factor Receptor (VEGF) and Snail expression, and suppressed tumor growth in vivo.

ONC206 exhibited an even greater potency in EOC models, enhancing ClpP expression and mitochondrial depolarization while inducing apoptosis more effectively than ONC201 [53].

**Figure 11:**
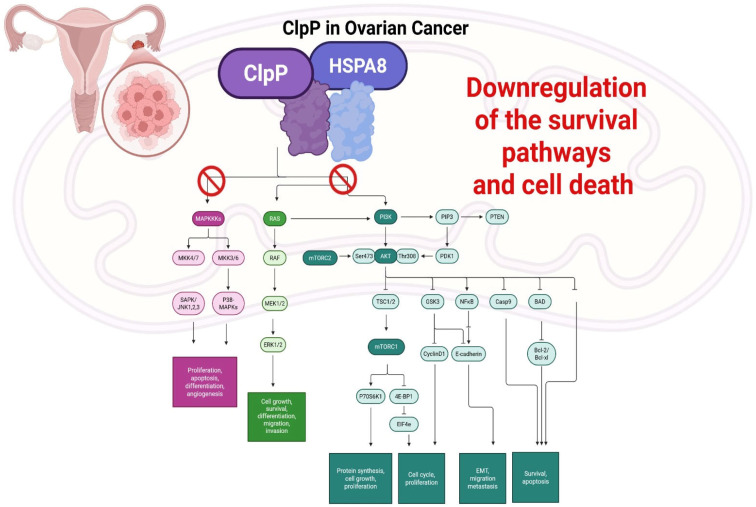
Schematic representation of ClpP–HSPA8 axis in EOC. HSPA8 destabilizes ClpP, promoting chemoresistance through enhanced mitophagy and mitochondrial resilience. ClpP activation by drugs disrupts key oncogenic signaling pathways, including MAPK/ERK and PI3K/AKT/mTOR, leading to downregulation of survival signaling, impaired proliferation, migration, and angiogenesis, and induction of apoptosis.

Thus, ClpP in EOC has a dual role: its downregulation confers resistance by enhancing mitophagy and mitochondrial resilience, while its activation triggers mitochondrial dysfunction and cell death. The discovery of the HSPA8-ClpP axis provides additional insight into stress adaptation mechanisms. Importantly, ClpP activators such as ONC201 and ONC206 not only exert cytotoxic effects but also inhibit pathways involved in metastasis, angiogenesis, and survival signaling. Given the poor prognosis of recurrent EOC and limited therapeutic options, targeting ClpP-mediated mitochondrial proteostasis represents a compelling strategy to overcome chemoresistance and improve patient outcomes [28] (Figure 11).

### 2.11. ClpP in Breast Cancer: Mitochondrial Stress, Senescence, and Metabolic Vulnerability

#### 2.11.1. Mechanism of Action

ClpP emerges as a mitochondrial checkpoint with subtype-specific implications in breast cancer. Its activation triggers senescence and immunogenicity in hormone receptor-positive tumor models such as BT474, MCF7, and HER2 cells, and induces metabolic collapse and stemness inhibition in triple-negative breast cancer (TNBC).

In BT474 cell line, short-term exposure to the ClpP activator ONC201 induces transient mitochondrial stress and ISR activation, marked by the upregulation of ATF4, CHOP, and GDF-15. However, prolonged ONC201 treatment leads to sustained ISR, mtDNA loss, G1/S cell cycle arrest, and the emergence of a senescence-like phenotype, characterized by downregulation of cyclin E and Cdk2 [54]. This stable arrest enhances tumor immunogenicity, particularly through increased susceptibility to natural killer (NK) cell-mediated killing, suggesting that ClpP activators may synergize with immunotherapies in this context.

In MCF7 cells, cisplatin induces the expression of ClpP, HSP60, and LONP1 via SIRT3, a mitochondrial deacetylase. SIRT3 silencing abolishes this mitochondrial stress response, evidencing a SIRT3–ClpP axis potentially involved in chemoresistance mechanisms [55].

Additionally, in HER2-overexpressing tumors, UPRmt activation has been observed in patient-derived samples. Elevated levels of ClpP, HSP60, and HSP10 correlate with higher tumor grade and appear to be driven by HER2/MAPK signaling, specifically through the JNK–CHOP–C/EBPβ axis. This demonstrates a direct mechanistic link between oncogenic pathways and ClpP-mediated mitochondrial stress signaling [55].

#### 2.11.2. Preclinical Data and Clinical Outlook

In TNBC, ClpP activation exerts potent antitumor effects by inducing metabolic dysfunction and enhancing sensitivity to combined therapies. Fennell et al. demonstrated that ClpP activators such as ONC201 and TR57 reduced specific essential matrix mitochondrial proteins, including OXPHOS and TCA cycle components, in a time-, dose-, and ClpP-dependent manner. These mitochondrial disruptions, absent in ClpP-deficient cells, led to metabolic collapse and inhibited proliferation of TNBC cells [55].

Furthermore, Lim et al. showed that ONC201 synergizes with trametinib in TNBC by promoting activation of caspase-3 and -7. In this study, ClpP and SOD2 expression levels predicted treatment response, while resistance was associated with elevated HER2_pY1248, PLK1, and phosphorylated Rb [56].

**Figure 12:**
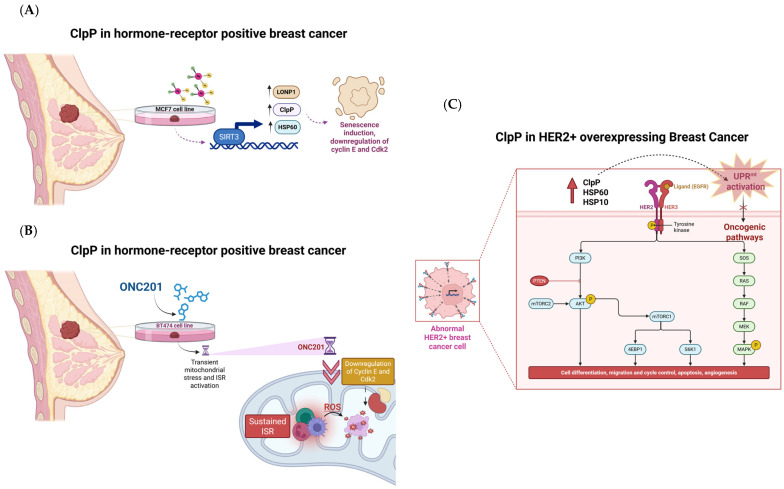
ClpP as a mitochondrial checkpoint in breast cancer subtypes. (**A**) In MCF7 cells, ClpP upregulation is linked to reduced SIRT3 expression, senescence induction, and downregulation of cyclin E/Cdk2; (**B**) In BT474 cells, ONC201 induces mitochondrial stress, ISR activation, ROS accumulation, and suppression of cyclin E/Cdk2; (**C**) In HER2+ breast cancer, ClpP and mitochondrial chaperones promote UPRmt and activation of oncogenic signaling pathways.

Additionally, Greer et al. demonstrated that ClpP modulators impair breast cancer stem cell (CSC) function by targeting glutamine-proline and one-carbon metabolism, resulting in NAD(P)+ depletion and oxidative stress. These compounds downregulated key metabolic enzymes (PYCR1/2, MTHFD2, SHMT2, TYMS) and suppressed the YAP and Myc oncogenic pathways via AMPK activation, processes known to support CSC maintenance and therapy resistance [57]. These data highlight the dual potential of ClpP modulators to induce mitochondrial stress and sensitize tumors to existing therapies, particularly in aggressive subtypes like TNBC and HER2-positive breast cancer (Figure 12).

### 2.12. ClpP in Brain Tumors: Mitochondrial Vulnerability in High-Grade Gliomas

#### 2.12.1. Mechanism

ClpP has emerged as a promising anticancer target following the discovery of ONC201, actually approved FDA to treat high grade glioma, and related small-molecule activators. While most studies have focused on hematological malignancies, particularly acute myeloid leukemia, where ClpP activation leads to mitochondrial dysfunction and selective cancer cell death, its role in solid tumors—especially brain tumors-remains comparatively underexplored. Only a limited number of research groups are investigating ClpP in the context of brain cancers, even though these malignancies often exhibit a strong dependence on mitochondrial metabolism. Given the central role of mitochondria in the survival and therapy resistance of gliomas such as diffuse midline gliomas (DMGs), targeting ClpP may offer a realistic therapeutic avenue.

Diffuse Intrinsic Pontine Glioma (DIPG), belonging to midline glioma (DMG), is primarily driven by H3K27M mutations in histone H3.1 or H3.3, which reprogram the epigenome through inhibition of PRC2, leading to global loss of H3K27me3 and oncogenic transcriptional activation. This molecular hallmark underpins the aggressive biology and therapy resistance of DIPG, distinguishing it from other pediatric gliomas [58,59].

Its activation, particularly via imipridones like ONC201, results in mitochondrial and epigenetic remodeling that underpins antitumor efficacy in preclinical and early clinical settings. Mechanistically, ONC201 targets dopamine receptor DRD2 and ClpP. Its mitochondrial effects include degradation of SDHA, IDH3B, COX4I1, and COX10, leading to oxidative stress, impaired OXPHOS, and suppression of TCA cycle function. These metabolic changes coincide with partial restoration of H3K27me3 and downregulation of cell cycle genes, aligning mitochondrial dysfunction with epigenetic reprogramming [60].

#### 2.12.2. Preclinical Data

Jazz Pharmaceuticals has recently announced FDA approval of ONC201 as Modeyso™, the first and only treatment for recurrent H3K27-altered DMGs. In early-phase clinical trials, ONC201 improved median overall survival in DIPG patients from 11.9 to approximately 20 months [59,60], including activity in non-midline H3 K27M-mutant gliomas. These results challenge anatomical restrictions of the DMG diagnosis and support a mutation-centric treatment rationale. Furthermore, gene expression profiling studies have begun to elucidate how radiotherapy reshapes the molecular landscape of DIPG/DMG. Understanding these radiation-induced transcriptional changes may enable more personalized and effective treatment approaches in pediatric brain tumors [59].

Building on the imipridone scaffold, recent efforts have led to the development of ONC201 analogs with enhanced potency and selectivity for ClpP. Among these, THX6 has shown high activation of ClpP (EC_50_ = 1.18 µM) and no significant affinity for DRD2, strong cytotoxicity in highly ONC201 resistant DIPG patient-derived cells, particularly SU-DIPG-VI (IC_50_ = 0.13 µM), and potent activity also in SU-DIPG-36, harboring the H3.1K27M mutation, and SU-DIPG-50, which carries the H3.3K27M variant [8]. THX6 also disrupted lipid homeostasis by altering fatty acid composition, with selective effects depending on cell line sensitivity. The compound stabilized ClpP in Cellular Thermal Shift Assay (CETSA) and exhibited comparable membrane permeability to ONC201, supporting its potential as a next-generation ClpP activator [8].

Additional support comes from harmaline, a β-carboline compound identified via virtual screening as a novel *h*ClpP modulator. Harmaline displayed moderate ClpP activation and cytotoxicity in DIPG-derived cell lines and neuroblastoma (GMB) spheroids, with stable *h*ClpP interactions and CNS penetrance, suggesting potential for optimization [1].

In GBM, ClpP activation synergizes with HDAC inhibitors such as panobinostat. Combined treatment induces apoptosis via downregulation of Bcl-xL and Mcl-1, abolishes mitochondrial respiration, and creates bioenergetic conflict that collapses tumor energetics [61]. Dopaminergic signaling also affects outcomes: DRD2 overexpression increases ONC201 sensitivity, but dopamine adaptation can confer resistance through p-AKT, XIAP, and c-FLIP upregulation [37]. Interestingly, DMG cells appear resistant to dopamine-mediated protection, reinforcing their imipridone sensitivity. In vivo efficacy of ONC201 in DIPG exceeds in vitro results, suggesting interference with tumor–neuron dopamine crosstalk.

**Figure 13:**
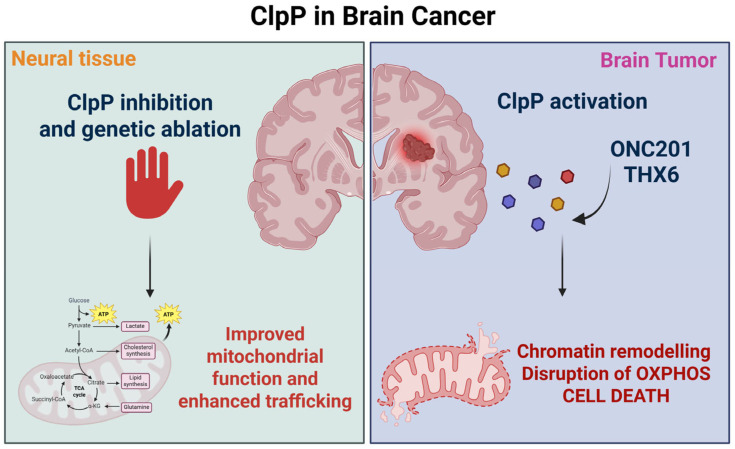
Context-dependent effects of ClpP modulation in the CNS. In neural tissue, ClpP inhibition or genetic ablation enhances mitochondrial function and trafficking, supporting neuronal integrity. In brain tumors such as DMG, activation of ClpP by compounds including ONC201 and THX6 induces mitochondrial proteotoxic stress, chromatin remodeling, disruption of oxidative phosphorylation (OXPHOS), and ultimately cancer cell death.

Beyond oncology, ClpP also modulates neural tissue homeostasis. In Dars2-deficient mouse models of mitochondrial encephalopathy, ClpP ablation preserved neuronal integrity, improved OXPHOS, and stabilized respiratory complex I and its supercomplexes [61]. Enhanced cristae morphology and mitochondrial trafficking were observed, particularly in Purkinje and hippocampal neurons, highlighting context-dependent roles of ClpP inhibition in CNS physiology (Figure 13).

#### 2.12.3. Clinical Outlook

Despite clinical benefit, resistance remains a critical challenge. Activation of PI3K/AKT signaling and NRF2-driven antioxidant pathways (e.g., NQO1) contribute to reduced response. Combining ONC201 with the PI3K/AKT inhibitor paxalisib (NCT03696355) resensitized resistant DIPG models and extended xenograft survival, confirming the rationale for ongoing clinical evaluation (NCT05009992) [60].

The importance of ClpP in CNS health is further emphasized by Perrault syndrome (PRLTS), caused by biallelic CLPP loss-of-function mutations, leading to hearing loss, ovarian insufficiency, and CNS white matter abnormalities [62]. Truncating mutations result in severe multisystem disease, whereas milder variants cause more limited phenotypes. This underscores that therapeutic modulation of ClpP must be context-specific, with activators in brain tumors and inhibitors in neurodegeneration.

Overall, ClpP is a key vulnerability in high-grade gliomas, linking mitochondrial metabolism, oxidative stress, and epigenetic control. Imipridones disrupt tumor energetics and drive apoptosis, particularly in DMG and GBM. Resistance via PI3K/AKT and antioxidant pathways can be overcome with rational drug combinations. At the same time, data from genetic and neurodevelopmental disorders caution that ClpP targeting must account for tissue-specific functions. Future trials should integrate metabolic and genetic biomarkers to refine ClpP-directed strategies in brain tumors [63,64,65,66].

## 3. Controversies and Knowledge Gaps in ClpP Biology and Therapeutic Targeting

ClpP/ClpX are widely expressed but show heterogeneity of expression at the tumor level and often non-prognostic associations, raising the question of whether ClpP abundance primarily marks mitochondrial stress rather than true oncogenic dependence. Consistent with this, therapeutic outcomes are highly context-dependent: neuronal cues such as dopamine can blunt the anticancer activity of imipridones in several solid tumors, while H3K27-altered DMG cells appear less protected—implicating tumor–neuron dopamine crosstalk as a potential gatekeeper of efficacy. In addition, imipridones are an example of polypharmacology targeting ClpP and DRD2 (possibly other pathways), and the relative contribution of each target varies depending on the experimental model under study; rigorous target-engagement controls are therefore essential to attribute responses to ClpP activation versus DRD2 antagonism in each biological context.

## 4. Conclusions

The selective activation of ClpP represents a novel and promising strategy in cancer therapy development. Across various malignancies, ClpP-targeted compounds have demonstrated the ability to impair tumor bioenergetics, overcome drug resistance, and induce selective cytotoxicity. This review highlighted the relevance of ClpP in a broad spectrum of cancers, including leukemia, gliomas, colorectal, breast, prostate, pancreatic, ovarian, and liver tumors. These effects are often mediated by mitochondrial collapse, metabolic reprogramming, and disruption of proteostasis.

ClpP emerges as a central regulator of multiple cancer-related processes, exerting its antitumor effects through the induction of mitochondrial stress, disruption of metabolic homeostasis, promotion of oxidative imbalance, and activation of apoptotic pathways. Its activation compromises mitochondrial proteostasis, ultimately impairing tumor cell viability and growth. These interconnected mechanisms position ClpP as a multifaceted therapeutic vulnerability across diverse tumor types. Importantly, ClpP functions within a broader mitochondrial quality control network, closely interacting with chaperones (e.g., HSP60) and other proteases (e.g., LONP1). These proteins cooperate to ensure proper protein folding, turnover, and stress response within mitochondria. Dysregulation of this proteostasis network contributes to cancer progression and therapeutic resistance. Therefore, investigating ClpP in the context of its interactions with HSP and LONP systems could uncover synergistic vulnerabilities and novel therapeutic opportunities.

Future research will need to address pharmacokinetic challenges, define tumor-selective ClpP activation thresholds, and explore combination strategies, including co-targeting mitochondrial proteostasis regulators, to translate these findings into effective clinical therapies. Moreover, the development of novel compounds based on diverse scaffolds will not only expand the repertoire of ClpP modulators but also provide deeper insights into the biology of ClpP and its context-dependent role in cancer.

Overall, ClpP represents a versatile and targetable vulnerability whose modulation, alone or in synergy with stress-adaptive pathways, holds significant promise for next-generation anticancer strategies.

## Figures and Tables

**Table 1:** Representative ClpP activators and inhibitors (highlighted in gray) [18,19] featuring different central core ring structures: therapeutic relevance and stage of development.

Compound [PMID]	Central Core Ring	Chemical Structure	Tumor Types [Mechanism]	Stage of Development
ONC201 [31021596] Modeyso™	2,4,6,7,8,9-hexahydroimidazo [1,2-*a*]pyrido[3,4-*e*]pyrimidin-5(1*H*)-one	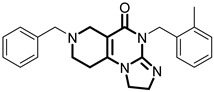	AML, CRC, prostate (NEPC), pancreatic, brain tumors (DMG H3K27-altered) [activator]	Clinical trials (phase III) FDA approved
IMP075 [36030831]	2,3,6,7,8,9-hexahydroimidazo[1,2-*a*]pyrido[3,4-*e*]pyrimidin-5(1*H*)-one	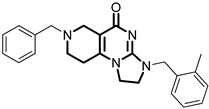	Colorectal cancer [activator]	Preclinical
NCA029 [38329974]	1,2,5,6-tetrahydropyridine-3-carboxamide	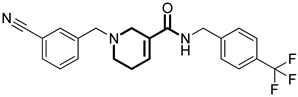	Tested in ClpP-related models [activator]	Preclinical
ONC206 [31021596]	hexahydroimidazo[1,2-*a*]pyrido[3,4-*e*]pyrimidin-5(1*H*)-one	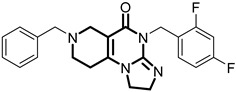	Hematological and solid tumors [activator]	Clinical trials (phase I, PNOC023)
ONC212 [31021596]	hexahydroimidazo[1,2-*a*]pyrido[3,4-*e*]pyrimidin-5(1*H*)-one	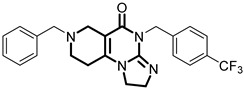	Hematological and solid tumors [activator]	Preclinical
TR57 [30783502]	5,6,7,8-tetrahydropyrido[4,3-*d*]pyrimidine-2,4(1*H*,3*H*)-dione	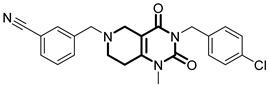	AML, other cancers [activator]	Preclinical
ZG36 [37352796]	1-acetylhexahydro-4*H*-pyrazino[1,2-*a*]pyrimidine-4,7(6*H*)-dione	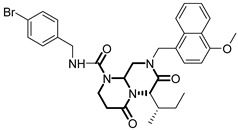	NSCLC, other tumors [activator]	Preclinical
ZK53 [37923710]	1-(piperazin-1-yl)ethan-1-one	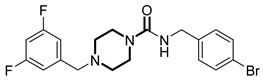	NSCLC [activator]	Preclinical
7k [38620134]	2-amino-5,6,7,8-tetrahydropyrido[4,3-*d*]pyrimidin-4(1*H*)-one	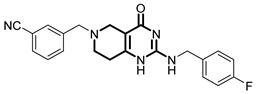	AML [activator]	Preclinical
ClpP-1071 [39574384]	2,4,6,7,8,9-hexahydroimidazo[1,2-*a*]pyrido[3,4-*e*]pyrimidin-5(1*H*)-one	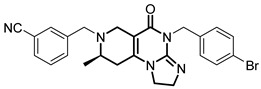	Not yet studied for tumor-specificity [activator]	Preclinical
THX6 [39973170]	tetrahydropyrido[4,3-*d*]pyrimidine-2,4(1*H*,3*H*)-dione	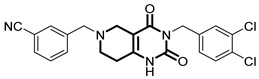	Not yet studied for tumor-specificity [activator]	Preclinical
A2-32-01 [21855356]	oxetan-2-one	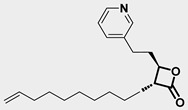	Tested in ClpP-related models [inhibitor]	Preclinical
TG53 [30109319]	phenylester	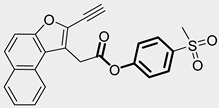	Tested in ClpP-related models [inhibitor]	Preclinical

## Data Availability

No new data were created or analyzed in this study. Data sharing is not applicable to this article.

## Abbreviations

The following abbreviations are used in this manuscript:

*h*ClpP	Human Caseinolytic Protease P
ClpX	Caseinolytic Protease X
UPRmt	Mitochondrial Unfolded Protein Response
OXPHOS	Oxidative Phosphorylation
GBM	Glioblastoma Multiforme
TCGA	The Cancer Genome Atlas
ROS	Reactive Oxygen Species
AML	Acute Myeloid Leukemia
ISR	Integrated Stress Response
HCC	Hepatocellular Carcinoma
NSCLC	Non-Small Cell Lung Cancer
TRAIL	Tumor Necrosis Factor-Related Apoptosis-Inducing Ligand
DMG	Diffuse Midline Glioma
ATP	Adenosine Triphosphate
CLL	Chronic Lymphocytic Leukemia
shRNA	Short Hairpin RNA
VEN	Venetoclax
BCR	Breakpoint Cluster Region
BCL-2	B-Cell Lymphoma-2
PDAC	Pancreatic Ductal Adenocarcinoma
ETC	Electron Transport Chain
2-DG	2-Deoxy-D-Glucose
AMPK	AMP-Activated Protein Kinase
DRD2	Dopamine Receptor D2
NASH	Non-Alcoholic Steatohepatitis
FAO	Fatty Acid Oxidation
SDHA	Succinate Dehydrogenase A
SDHB	Succinate Dehydrogenase B
ECH1	Enoyl-Coa Hydratase 1
COX5A	Cytochrome C Oxidase Subunit 5A
MAD1L1	Mitotic Arrest Deficient 1 Like 1
EB1	End Binding 1
DR5	Death Receptor 5
PARP	Poly (ADP-ribose) polymerase
CRC	Colorectal Cancer
PCa	Prostate Cancer
CRPC	Castration-Resistant Prostate Cancer
NEPC	Neuroendocrine Prostate Cancer
AR	Androgen Receptor
NED	Neuroendocrine Differentiation
SOX2	SRY-box transcription factor 2
POU3F2	POU class 3 homeobox 2
SCLC	Small Cell Lung Carcinoma
SNPs	Single Nucleotide Polymorphisms
SCC	Squamous Cell Carcinoma
LUSC	Lung Squamous Cell Carcinoma
DDR	DNA Damage Response
EOC	Epithelial Ovarian Cancer
NDUFA12	NADH:ubiquinone oxidoreductase subunit A12
EMT	Epithelial–Mesenchymal Transition
ER	Endoplasmic Reticulum
VEGF	Vascular Endothelial Growth Factor Receptor
TNBC	Triple-Negative Breast Cancer
TCA	Tricarboxylic Acid Cycle
NK	Natural Killer
CSC	Cancer Stem Cell
DMG	Diffuse Midline Gliomas
DIPG	Diffuse Intrinsic Pontine Glioma
IDH3B	Isocitrate Dehydrogenase (NAD(+)) 3 Non-Catalytic Subunit Beta
COX4I1	Cytochrome c oxidase subunit 4 isoform 1
CETSA	Cellular Thermal Shift Assay
HDAC	Histone Deacetylase
Bcl-xL	B-cell lymphoma-extra large
Mcl-1	Induced myeloid leukemia cell differentiation protein
NQO1	NAD(P)H dehydrogenase [quinone] 1
PRLTS	Perrault syndrome
